# A phase I dose-escalation trial of stereotactic ablative body radiotherapy for non-spine bone and lymph node metastases (DESTROY-trial)

**DOI:** 10.1186/s13014-018-1096-9

**Published:** 2018-08-20

**Authors:** Carole Mercier, Piet Dirix, Paul Meijnders, Peter Vermeulen, Steven Van Laere, Hilde Debois, Philippe Huget, Dirk Verellen

**Affiliations:** 1 Department of Radiation Oncology, Iridium Cancer Network, Wilrijk, Antwerp Belgium; 2 Translational Cancer Research Unit, Oncologisch Centrum GZA, Wilrijk, Antwerp Belgium; 3 University of Antwerp, Molecular Imaging, Pathology, Radiotherapy & Oncology (MIPRO), Edegem, Antwerp Belgium; 4 Departement of Radiotherapy, UZ Brussel, Vrije Universiteit Brussel, Jette, Belgium

**Keywords:** Stereotactic ablative radiotherapy, Non-spine bone metastases, Lymph node metastases

## Abstract

**Background:**

In an oligometastatic setting, metastasis-directed treatment could render patients disease free, possibly for a protracted interval. Stereotactic ablative radiotherapy (SABR) is one of the treatment modalities that can be offered to these patients. In addition, the radiobiological qualities of SABR are promising for the use in perceived radioresistant tumours. There is also emerging evidence that SABR can stimulate the immune response, and a specific therapeutic window may exist for the optimal use of radiotherapy as an immune adjuvant. However, when SABR is considered for non-spine bone or lymph node metastases, the optimal fractionation schedule is not yet known.

**Methods:**

The DESTROY-trial is a non-randomized prospective phase I trial determining a regimen of choice for patients with non-spine bone and lymph node metastases. A total of 90 patients will be included in three different treatment regimens. They will be offered stereotactic ablative radiotherapy in 5, 3 or 1 fractions. Dose-limiting toxicity will be recorded as primary endpoint. Acute and late toxicity, local response and local recurrence, and progression-free survival are secondary endpoints. Liquid biopsies will be collected throughout the course of this study from the second fractionation schedule on.

**Discussion:**

Despite its almost universal use in (oligo-)metastatic patients, the level of evidence supporting radical local treatment in general, and stereotactic radiotherapy in particular, is low. This prospective phase I trial will evaluate different SABR regimens for metastases and the differences in immune-stimulatory effects.

**Trial registration:**

The Ethics committee of the GZA Hospitals (B099201732915) approved this study on 05/07/2017. Amendment for translational research was approved on 06/02/2018. Trial registered on Clinicaltrials.gov (NCT03486431) on 03/04/2018 – Retrospectively registered.

## Background

The term oligometastatic disease describes an intermediate state of cancer spread between localized disease and widespread metastases. It is the state in which a patient presents with a limited number of synchronous or metachronous metastases, with the primary tumour either controlled or not. Weichselbaum and Hellman theorized that before development of widespread metastases, malignant cells harbour restricted metastatic potential and occupy only a limited number of sites apart from the primary tumour [1]. Theoretically, aggressive metastasis-directed therapy (MDT) during this time could delay the need to start systemic therapies and/or prolong progression-free survival. The concept of oligoprogression refers to the situation of disease progression in a limited number of locations after an initial response to systemic treatment. This situation is getting more common in the setting of highly innovative systemic therapies, where metastasis-directed therapy could eradicate disease that does not respond to these pharmaceuticals and thus delay the need to change systemic therapy [2].

Stereotactic ablative body radiotherapy (SABR) is one of the MDTs that can be offered to patients with oligometastatic or oligoprogressive disease, who often present with minimal or no associated symptoms [3, 4]. SABR is defined as the precise delivery of highly conformal and image-guided hypofractionated external-beam radiotherapy to extracranial tumours, typically in a single or a few fraction (s), with doses at least biologically equivalent to a conventional radical course of treatment [5]. However, since this is a relatively new technique, information on the optimal scheduling is lacking. Even prospective randomized trials on SABR for oligometastatic disease typically allow different fractionation schedules to be used [6]. This is especially true for non-spine bone and lymph node metastases, where the literature is scarce to non-existent and many different schedules are used, even within a single center [7, 8].

The radiobiological qualities of SABR are potentially useful when treating perceived radioresistant tumours like renal cell carcinoma or malignant melanoma. Radioresistance may be overcome with dose escalation, particularly by increasing the dose per fraction, because of a direct effect on vasculature and cell membranes, rather than through oxygen-dependent DNA damage [9].

Apart from the direct effect of SABR on metastases, there is also emerging evidence that this treatment can improve disease control outside the irradiated volume by stimulating the immune response. This could potentially lead to so-called “abscopal” effects, i.e. response in non-irradiated lesions. A variety of mechanisms, such as increasing TLR4 expression on dendritic cells, increasing priming of T cells in draining lymph nodes, and increasing tumor cell antigen presentation by dendritic cells, is responsible for this effect [10]. Again, it is not clear which fractionation schedule elicits the most robust immune response. For instance, in combination with anti-CTLA-4 immunotherapy, different radiation regimens in two carcinoma models growing in syngeneic mice were compared [11]. Marked differences in induction of tumor-specific T cells and of an abscopal effect were observed. Each regimen had similar ability to inhibit the growth of the irradiated tumor when radiation was used alone. The addition of anti-CTLA-4, however, caused complete regression of the majority of irradiated tumours and an abscopal effect in mice receiving a hypofractionated regimen (3 fractions of 8 Gy) but not in mice treated with a single dose of 20 Gy. An additional fractionated regimen (5 fractions of 6 Gy) was tested, which showed intermediate results. This indicates that a specific therapeutic window may exist for the optimal use of radiotherapy as an immune adjuvant.

An interesting measure of DNA damage in circulating tumor cells (CTCs) is γ-H2AX, a biomarker for radiation-induced DNA double-strand breaks [9]. Apparently, DNA exonuclease Trex1 is induced by radiation doses above 12–18 Gy in different cancer cells, and attenuates their immunogenicity by degrading DNA that accumulates in the cytosol upon radiation. Cytosolic DNA stimulates secretion of interferon-b by cancer cells following activation of the DNA sensor cGAS and its downstream effector STING. Repeated irradiation at doses that do not induce Trex1 amplifies interferon-b production, resulting in recruitment and activation of Batf3-dependent dendritic cells [10]. This effect is essential for priming of CD8+ T cells that mediate systemic tumour rejection (abscopal effect). These data suggest a link between the immune-stimulatory effects of radiation and the DNA damage response.

It therefore seems an opportune moment to compare the most commonly used stereotactic regimens regarding toxicity and efficacy.

## Methods/design

The DESTROY-trial is a non-randomized phase I trial evaluation SABR as treatment for non-spine bone and lymph node metastases. After giving informed consent, patients are included in the DESTROY-trial, which was approved by the Ethics Committee of the GZA Hospitals.

### Objectives

- Primary endpoint
  - To determine the maximum tolerated dose (MTD): the maximal tolerated dose will be defined as the dose level below which at least 10 patients present with a dose-limiting toxicity (DLT) at 6 months after SABR.
    DLT will be defined as any acute grade 3 or 4 toxicity. The DLT observation window is 24 weeks after SABR was delivered. The severity of toxicity will be graded using the Common Terminology Criteria for Adverse Events (CTCAE), version 4.03. For events not reported in the CTCAE version 4.03, the investigator will use the grade or adjectives reported in Table 1.
- Secondary endpoints
  - To assess acute and late toxicities following SABR: acute as well as late toxicity will be assessed using the CTCAE version 4.03.
  - To evaluate local response (LR) and local failure (LF): LR will be evaluated by the response evaluation criteria in solid tumors version 1.1 (RECIST v1.1). LF will be scored as an event if an irradiated lesion shows an increase in size of ≥20%, according to RECIST v1.1.
  - To evaluate progression-free survival (PFS): PFS is defined as the time from inclusion to documented disease progression according to RECIST v1.1 or any other clinically relevant definition (e.g. biochemical progression in prostate cancer) or death from any cause.

**Table 1:** grade of toxicity

Grade	Adjective	Description
Grade 1	Mild	Does not interfere with patient’s usual function
Grade 2	Moderate	Interferes to some extent with patient’s usual function
Grade 3	Severe	Interferes significantly with patient’s usual function
Grade 4	Life-threatening	Results in a threat to life or in an incapacitating disability
Grade 5	Death	

### Inclusion criteria

- Patients ≥18 years old with histologically confirmed malignancy.
- Patients with radiosensitive malignancy (e.g. breast, prostate …) and oligometastases (i.e. ≤ 3 metastases) OR patients with radioresistant malignancy (e.g. renal cell carcinoma, melanoma, …) and an unlimited number of metastases.
- In an oligometastatic setting, all visible metastases should be treated with MDT. For patients with more than 3 radioresistant metastases, only the symptomatic lesion will be treated with SABR.
- Metastatic lesion must be visible on CT and < 5 cm in largest diameter.
- Patients with ECOG Performance Status ≤1.
- Patients who have received the information sheet and signed the informed consent form.
- Patients must be willing to comply with scheduled visits, treatment plan, and other study procedures.
- Patients with public health insurance coverage.

### Exclusion criteria

- Patients with life expectancy < 6 months.
- Patients with previous radiotherapy to the metastatic area excluding stereotactic re-irradiation to the required dose level.
- Patients with significantly altered mental status or with psychological, familial, sociological or geographical condition potential hampering compliance with the study.
- Individual deprived of liberty or placed under guardianship.

### Interventions

A minimum of thirty patients will be included for each dose level. The different treatment regimens and their biological effective doses (BED_10Gy_) and equivalent dose in 2 Gy per fraction (EQD_2Gy_) are presented in Table 2. Calculations for BED_10Gy_ and EQD_2Gy_ are made using an alpha/beta ratio of 10 and 2, respectively. An interval of at least 24 weeks from the first patient treatment to the next patient treatment at each dose level will be respected. In the meantime, more patients will be included in the previous dose level, in an effort to establish the secondary endpoints. In case 1 to 5 patients present with DLT at 6 months after SABR, 30 additional patients will be included at the same dose level.

**Table 2:** Fractionation schedules

Level	Fractions	Interval	Dose/fraction	Total	BED_10Gy_	EQD_2Gy_
1	5	48 h	7	35	59.5	78.75
2	3	48 h	10	30	60.0	90
3	1	NA	20	20	60.0	110

### Time schedule and follow-up

The aim is to recruit the necessary number of patients within a timeframe of 48 months. Follow-up of these patients will be life-long in order to correctly estimate the secondary endpoints (Table 3). Patients are seen centrally by the clinical research physician at 3 and 6 months after SABR. At 6 months, repeated imaging is performed in the study to assess local control. All imaging is considered standard and should minimally include a CT of the irradiated lesion (s) but might also include MRI and/or PET-CT (with whatever relevant tracer) if standard for that malignancy. Reports on acute toxicity will be expected within 2 months after closure of the trial.

**Table 3:** Time schedule

	TRIAL PERIOD						FOLLOW-UP		
	Screening visit	Pre-study	During study				Year 1	Year 2	Year 3 till ∞
			Every SABR fraction	Last day of SABR	3 months after SABR	6 months after SABR	Every 3 months	Every 6 months	yearly
Informed consent	x								
Clinical examination	x			x	x	x	x	x	x
Registration of toxicity		x		x	x	x	x	x	x
Registration of QoL		x		x	x	x	x	x	x
Blood sample		x	x	x	x	x			
Imaging						x			

### Treatment details

#### Localization, simulation and immobilization

All patients will be immobilized in a comfortable and appropriate position to irradiate the metastatic lesion (s). Support and/or immobilization devices can be used to increase patient comfort or to ensure set-up reproducibility. The planning CT scan should be acquired with the patient in the same position and using the same immobilization/support device (s) as for treatment. Planning CT scan (without intravenous contrast for bone metastases but with intravenous contrast for lymph node metastases, unless contraindicated) will be done at < 3 mm intervals encompassing the region of interest with sufficient margin for treatment planning, a typical scan length should extend at least 10 cm superior and inferior beyond the treatment field borders.

#### Target contouring

The Gross Tumour Volume (GTV) will consist of the metastatic lesion (s) as visualized on CT. All available diagnostic imaging (PET-CT, MRI) is used in order to delineate the target structures as accurate as possible. No Clinical Target Volume (CTV) will be delineated. The Planning Target Volume (PTV) will be created by using a 3-dimensional margin on the GTV to allow for daily set-up variance and organ motion. Margins depend on the site irradiated with 3 mm margins for bony lesions and 5 mm for lymph nodes.

#### Organs at risk

Organs at risk (OAR) are contoured as visualized on the planning CT. The OAR depend on the localization of the metastases and should at least include all OAR (lying within the scanned range on the planning CT scan) for which dose constraints are described in the report of the AAPM task group 101 [12]. A Planning Organ at Risk Volume (PRV) expansion will be added to the OAR for setup uncertainty or organ motion. For mediastinum, liver, heart and kidney a PRV margin of 5 mm should be used. All dose constraints apply to this PRV. It is strongly recommended that dose constraints be not exceeded. If a dose constraint cannot be achieved due to overlap of the target with an OAR, the target coverage can be compromised in order to meet the constraint.

#### Treatment planning & prescription

SABR (static or rotational) treatment planning will be dependent the localization of the metastasis. Prescribing, recording and reporting of the doses will be consistent with the recommendations in Report 91 of the International Commission on Radiation Units and Measurements (ICRU) [13]. Treatment will be prescribed to the periphery of the target, i.e. 80% of the dose should cover 90% of the PTV. A dose inhomogeneity in the PTV overlapping with a PRV is allowed but 90% of the GTV should receive the prescribed dose. Maximum PTV dose up to 160% is allowed but all dose > 105% should lay within the PTV. Dose fall-off outside the PTV extending into normal tissue structures must be rapid in all directions and one should target a dose fall-off of 50% off the prescribed dose within 3 cm outside the PTV. The OAR dose constraints will be in accordance with the recommendations from the report of the AAPM task group 101 [12].

#### Delivery and verification

Treatment will be delivered with 6–10 MV photons of a linear accelerator using cone-beam CT set-up and on-line correction of patient’s position. No other radiotherapy than photon therapy is permitted. The same position and immobilization/support device (s) as used in the planning CT scan should be utilized.

#### Interruptions

Treatment interruptions will usually not be necessary and should be kept to a minimum. Rarely, (unexpected) toxicities or non-treatment-related events may require interruption of treatment at the discretion of the investigator. Every effort should be made to limit treatment interruptions to a maximum of 5 days (including weekends and national holidays). In case of treatment interruption, Treatment should be completed to the prescribed doses. The total number of fractions and the overall treatment time should be reported.

### Translational research

#### Required samples

From the second dose level on (3x10Gy), liquid biopsies will be collected throughout the course of this study for biobanking. The liquid biopsy in this study encompasses pheripheral blood samples (1 × 9 mL ethylenediaminetetraacetic acid (EDTA) tube and 1 × 9 mL cellular preparation tube (CPT)), to be taken at simulation, immediately after each fraction, approximately 2 weeks after the last fraction, and at 3 and 6 months follow-up.

#### Assessment of circulating cytokines

One EDTA blood tube generally yields 4 mL of plasma, which can be split in half for circulating free deoxyribonucleic acid (cfDNA) analysis (vide infra) and for the measurement of protein concentrations of circulating cytokines. This latter can be done using Luminex assays, and requires and input volume of 100 μL per assay, allowing 20 cytokines to be profiled using 2 mL of plasma. The plasma must be kept at − 80 °C. Under these conditions, most cytokines are stable for up to 2 years under the premises that freeze-thaw cycles are avoided [11].

#### cfDNA for shallow whole genome sequencing

For cfDNA low-pass whole genome sequencing, cfDNA first needs to be extracted from plasma samples with a typical starting volume of 1 mL. The cfDNA concentrations from 1 mL of plasma, in a final elution volume of 50 μL, are highly variable and depend on tumour burden (range 0.2 ng/μL to 62.8 ng/μL). Hence the calculated cfDNA yield from 1 mL of plasma ranges from 10 ng to 3140 ng. For low-pass whole genome sequencing using the Thru-PLEX™ DNA-seq Library Kit, 2 ng of cfDNA is required, suggesting that 1 mL of plasma should be sufficient in most cases. To avoid patient drop-out due to insufficient starting material, biobanking 2 mL of plasma aliquoted in units of 400 μL at -80oC is advisable. [12].

#### Flow cytometry analysis of immune cells

Peripheral blood mononuclear cells (PBMC) can be isolated from heparinized venous blood by centrifugation on a Ficoll-Hypaque gradient within 4 h of venepuncture. The PBMCs can cryopreserved in liquid nitrogen in heat-inactivated foetal bovine serum (FBS) supplemented with 10% dimethyl sulphoxide (DMSO) until analysis. Upon analysis, cells are thawed by submersion at 37° for 1–2 min and resuspended in a medium containing Iscove’s Modified Dulbecco’s Medium supplemented with 20% FBS and 1% glutamine [13].

### Statistical analysis

#### Sample size

For each dose level, a minimum of 30 patients will be included. The maximal tolerated dose will be defined as the dose level below which at least 10 patients present with a DLT at 6 months after SABR.

#### Data analysis

All data will be prospectively collected. Electronic case report forms will be used; database will be excel-based and Access-based. Statistics will be carried out using the latest version of SPSS. The primary endpoint MTD will be evaluated at 6 months after the end of SABR. The incidence of acute and late toxicity will be recorded. Chi-square test will be used to find out the significance of results. Disease free survival will be calculated using Kaplan Meier actuarial analysis. Survival times are defined from the date of start of the treatment until an event or last follow up.

## Discussion

Oligometastatic disease is a state in which prolonged progression-free survival resulting from metastasis-directed therapy may be achievable in a subset of patients. For instance, in the pivotal trial by Ost and colleagues, around 20% of oligometastatic prostate cancer patients remained disease-free at 2-years follow-up after only MDT [14]. The role of SABR in the setting of metastases is still emerging but the early results for LC are promising. Most clinical trials evaluating efficacy and safety of this technique are retrospective. Furthermore, most have included various radiation dosing, and especially for non-spine bone and lymph node metastases the evidence on which fractionation schedule to use, is scarce. A phase III clinical trial is currently underway, which is comparing schemes of single doses of 24 Gy to hypofractionated doses of three 9 Gy fractions for different types of metastases, including non-spine bone and lymph node metastases [15].

For lymph node metastases of various histologies, the use of SABR generates high LC levels of 80% and more, with acceptable toxicity [16, 17]. However; various fractionation schemes are described in the literature, which range between single doses of 16 Gy and higher to schemes of 10 fractions of 5 Gy [16–23].

When SABR is used for treating non-spine bone metastases, the results are also encouraging. A one-year local control rate of more than 90% is reported in a restrospective analysis of 106 non-spine bone metastases treated with SABR, which is comparable to the results of a prospective study of 85 non-spine bone metastases treated with SABR [8, 24]. In the meantime, these studies showed no grade 3 or greater late toxicities, and very few grade 1 or grade 2 pain flare. A review of Bedard on the use of SABR for non-spine bone metastases reported similar results, but due to the lack of consistency in endpoint definitions, it was difficult to compare outcomes across trials [25].

SABR is also often used for treating metastases from radioresistant tumors like renal cell carcinoma or malignant melanoma. Radioresistance may be overcome with dose escalation, particularly by increasing the dose per fraction [9]. A systematic review of Kothari ea. evaluating the use of SABR found excellent LC rates and low rates of toxicity for intracranial and extracranial metastatic renal cell carcinoma [26]. They reported a weighted one-year LC rate of 86% and grade 3–4 toxicities ranging from 0 to 4%. In the setting of oligometastasized malignant melanoma, there is less clinical data available. Nevertheless, a prospective study showed high rates of complete response and durable metastasis control with SABR for extracranial metastases of malignant melanoma, including bone and lymph node metastases [27]. Both for malignant melanoma and for renal cell carcinoma, LC rates achieved are comparable to those obtained with SABR for other histologies, suggesting a dominant mechanism of in vivo tumor ablation that overrides intrinsic differences in cellular radiosensitivity between histologic subtypes [28].

The concept of a risk-adapted approach for SABR fractionation regimens, using smaller fraction sizes for tumor locations that overlap with organs at risk for toxicity, has gained widespread acceptance. This concept is based on the assumption that fractionation relatively decreases the EQD2 for normal tissues. The results of this dose escalation trial, with increasing dose per fraction, can help to improve the risk-adapted approach for non-spine bone and lymph node metastases. Also important in this regard is the alpha/beta ratio of 10 used in the BED calculations, which is an estimate of the general alpha/beta ratio of tumor metastases. However, the available clinical data support the use of a lower alpha/beta for renal cell carcinoma, breast or prostate cancer, which would lead to a higher BED.

To keep the study protocol accessible to most metastasized patients, there is no limitation on the use of concurrent systemic treatment, but this gives a possible increase in acute or late toxicity.

Even though the data are still not sufficient to be able to routinely recommend SABR, the high LC rates and low toxicity rates observed after SABR in patients with oligometastatic disease and in patients with radioresistant metastases justifies continued exploration of strategies for ablative dose delivery within research protocols. To date, we do not have any clear results that demonstrate the benefit of one type of dose or fractioning over another. The aim of this prospective phase I trial is to establish the optimal regimen of SABR for non-spine bone and lymph node metastases, regarding acute and late toxicity as well as efficacy.

## Abbreviations

BED: Biologically effective dose
cfDNA: Circulating free DNA
CPT: Cellular preparation tubes
CTC: Circulating tumor cell
CTCAE: Common toxicity criteria for adverse events
CTV: Clinical target volume
DLT: Dose-limiting toxicity
DMSO: 10% dimethyl sulphoxide
EDTA: Ethylenediaminetetraacetic acid
EQD2: Equivalent dose in 2Gy per fraction
FBS: Foetal bovine serum
GTV: Gross tumour volume
LC: Local control
LR: Local recurrence
MDT: Metastasis-directed therapy
MTD: Maximum tolerated dose
OAR: Organ at risk
PBMC: Peripheral blood mononuclear cells
PFS: Progression-free survival
PRV: Planning organ at risk volume
PTV: Planning target volume
RECIST: Response evaluation criteria in solid tumours
SABR: Stereotactic ablative radiotherapy
